# First detection and genotyping of *Giardia intestinalis* in stool samples collected from children in Ghazni Province, eastern Afghanistan and evaluation of the PCR assay in formalin-fixed specimens

**DOI:** 10.1007/s00436-017-5529-4

**Published:** 2017-06-13

**Authors:** Anna Lass, Panagiotis Karanis, Krzysztof Korzeniewski

**Affiliations:** 1 Department of Tropical Parasitology, Institute of Maritime and Tropical Medicine in Gdynia, Medical University of Gdansk, 9b Powstania Styczniowego Str, 81-519 Gdynia, Poland; 2 State Key Laboratory of Plateau Ecology and Agriculture, Center for Biomedicine and Infectious Disease, Qinghai Academy of Animal Sciences and Veterinary Medicine, Qinghai University, Xining, 1#Wei’er Road, Qinghai Biological Scientific Estate Garden, Xining, 810016 People’s Republic of China; 3 Epidemiology and Tropical Medicine Department in Gdynia, Military Institute of Medicine in Warsaw, Grudzinskiego St. 4, 81-103 Gdynia, Poland

**Keywords:** *Giardia*, Nested PCR, Real-time PCR, Assemblages, Children, Afghanistan

## Abstract

It is estimated that faecal-orally transmitted diseases are common in Afghanistan, as a consequence of poor hygienic standards of life and widespread contamination of water and food with both human and animal faeces. However, there is little information in the literature concerning infections caused by intestinal parasites in the Afghan population. In this study, we report the occurrence of *Giardia intestinalis* assemblages (A and B) in formalin-fixed stool samples collected from 245 Afghan schoolchildren living in Ghazni Province in eastern Afghanistan. Detection of the parasite’s DNA and genotyping was performed using real-time PCR, specific to the β-giardin gene of *G. intestinalis*. Positive results were recorded in 52 (21.2%) samples. Genotyping was successful in 39 faecal samples and showed the predominance of assemblage B of *G. intestinalis* in this population (15 assemblage A and 24 assemblage B). Co-infection with both genotypes A and B was detected in four samples. Additionally, we evaluated the effect of 10% buffered formalin fixative on the detection of *G. intestinalis* DNA using real-time PCR and nested PCR characterised by different lengths of PCR products (74 and 479 bp, respectively). The human faeces containing the *Giardia* cysts were tested for 16 weeks. Amplification of *G. intestinalis* DNA with real-time PCR was possible up to 6 weeks of preservation of stool sample in formalin, compared to only 2 weeks with nested PCR. This suggests that real-time PCR is a more suitable tool in cases where stool samples have to be kept in formalin for longer periods of time.

## Introduction

*Giardia intestinalis* is a protozoan parasite belonging to the *Giardia* genus which causes gastrointestinal diseases in humans and animals worldwide (Karanis and Ey 1998; Smith et al. 2007; Reynolds et al. 2008; Ryan and Cacciò 2013). It is a species complex consisting of eight genetically distinct genotypes (assemblages A–H) which differ in terms of host specificity, of which two (A and B) have been commonly reported to be associated with human infections (Homan 1992; Mayrhofer 1995; Nash 1995; Andrews et al. 1998; Karanis and Ey 1998; Thompson 2000). However, new reports in the 10 last years from various countries regarding the assemblages’ distribution in humans and animals shed new light on, and pose new questions about, the zoonotic/anthropozoonotic character and transmission of the *Giardia* assemblages. The cattle-specific or livestock-specific assemblage E was detected in humans in Brazil (Fantinatti et al. 2016) and in Egypt (Foronda et al. 2008; Abdel-Moein and Saeed 2016); this probably demonstrates a new anthropozoonotic route of *Giardia* transmission. Microscopic investigation is not suitable for differentiation of genotypes or assemblages of this parasite; molecular methods are required (Karanis and Ey 1998; Xiao and Fayer 2008).

Giardiasis represents a significant public health problem worldwide. Ingestion of *Giardia* cysts can lead to the development of apparently sometimes asymptomatic infection. Patients may suffer from severe diarrhoea, abdominal cramps and nausea, among other symptoms of giardiasis; an acute phase of the disease, commonly lasting a few weeks, may develop into a chronic infection (Wolfe 1992; Farthing 1996; Adam 2001; Lebwohl et al. 2003). In humans, the prevalence of *Giardia* infections may vary between 2 and 5% in industrialised countries and 20 and 30% in developing countries (Thompson and Monis 2004). This parasite is transmitted mainly through contaminated water and food (faecal-oral route) (Karanis et al. 2007; Baldursson and Karanis 2011; Efstratiou et al. 2017). To date, at least 913 outbreaks associated with the waterborne transmission of protozoan parasites have been reported, of which *G. intestinalis* has been responsible for at least 340 (37.2%) (Karanis et al. 2007; Baldursson and Karanis 2011; Efstratiou et al. 2017).

Afghans living in poor socioeconomic conditions are believed to constitute a community with a high rate of intestinal parasitic infections. In Afghanistan, diseases caused by faecal-orally transmitted pathogens are common, as a consequence of poor hygienic standards and widespread contamination of water and food with both human and animal faeces. However, reports concerning population morbidity rates are often unconfirmed by laboratory tests (Elyan et al. 2014). In the literature, there is little information concerning infections caused by intestinal parasites in the Afghan population. Usually, studies are performed among refugees in the USA and Europe (Harp 2003; Hotez et al. 2007); only a few parasitological investigations have been performed in Afghanistan. In 2002, researchers from the Medical Parasitology Laboratory of the Central Institute of the Bundeswehr Medical Service Koblenz in Germany examined 217 local workers from the international military base, showing that 64% of them were infected with intestinal helminths and protozoa (Scheid and Thoma 2004). In 2003, the World Health Organisation performed a screening examination of stool samples taken from 1001 children, aged 8–15, in four provinces of the country, and confirmed the occurrence of intestinal helminthiases in 47% of the children, with a predominance of *Ascaris lumbricoides* (Gabrielli et al. 2005).

Since 2002, representatives of the military health service of the Polish Armed Forces have been stationed in eastern Afghanistan. Within the framework of humanitarian aid (project titled ‘Capacity building of health care system in Ghazni Province’, funded by the Polish Ministry of Defence), the Polish medical staff, mandatorily assigned to a Forward Operating Base in Ghazni, collected biological samples for parasitological research from patients of the provincial hospital and children attending the primary and secondary schools. The aim of this action was to prepare the scheme of deworming for the local population. Based on a microscopic screening study of this material, we showed a high prevalence of intestinal parasites in these populations (Korzeniewski et al. 2014, 2015a,b, 2016, 2017). Examination of 777 children hospitalised in the Ghazni Province Hospital performed in 2012–2013 showed the presence of intestinal parasitic infection in 40.2% of them, with a predominance of *A. lumbricoides* and *G. intestinalis* (17.2 and 16.7%) (Korzeniewski et al. 2014). Parasitological examination, conducted in 2013–2014, of stool samples collected from 1369 healthy children showed a 39% parasitic infection rate, including nematode, cestode and protozoan infections with prevalences of 23.8, 8.6 and 16.7%, respectively (Korzeniewski et al. 2015b).

The aim of the present study was to estimate the occurrence of *G. intestinalis* assemblages in formalin-fixed stool samples collected from Afghan schoolchildren living in Ghazni Province, eastern Afghanistan, using real-time PCR. Additionally, in this study, we made an attempt to evaluate the effect of 10% buffered formalin fixative and the influence of different times of incubation on the detection of *G. intestinalis* DNA from stool specimens using two assays: real-time PCR and nested PCR characterised by different lengths of PCR products.

## Material and methods

### Evaluation of the detection limit of *G. intestinalis* in human faeces using molecular methods depending on the duration of fixation in formalin

Prior to the investigations of *Giardia* strains in the Afghan schoolchildren, we evaluated the effect of 10% buffered formalin fixative on the detection of *G. intestinalis* DNA using real-time PCR and nested PCR. Human faecal sample containing cysts of *G. intestinalis*, selected from a population of polish children investigated previously in the Epidemiology and Tropical Medicine Department in Gdynia, Military Institute of Medicine in Warsaw for the presence of intestinal parasites (Korzeniewski et al. 2016), was used in the performed experiments. Light microscopy was used to confirm the presence of *G. intestinalis* cysts as well as to count them. The concentration of cysts in the selected sample was 440 per 1 μL of stool. This material was placed in a plastic container with 10% buffered formalin and stored at room temperature for 16 weeks. Every 2 weeks, two 0.1-g and two 0.6-g formalin-fixed stool samples were taken and investigated using two different molecular assays designed to detect *G. intestinalis* DNA, as described below. All negative samples from both series were retested with the use of a *G. intestinalis* reference DNA (DNA extracted from the selected faecal sample containing *Giardia* cysts prior to fixation in formalin as well as positive control used in molecular studies of Afghan material) to ensure the formalin inhibition effect on the samples tested. Material used for this test was different from the studied material (faecal samples collected from Afghan children) because the required procedures (especially DNA extraction and PCR) could not be performed in a military base. The main aim of the test was to check the general influence of 10% formalin on molecular detection of *Giardia* spp. in faecal material, depending on the duration of fixation, in order to decide which method, real-time PCR or nested PCR, would be more suitable for investigating material collected in Afghanistan.

### Study population of schoolchildren and material

In total, 245 Afghan children, aged 7–18, attending the Share Kona and Khuija Ali high schools (most students in both schools) in Ghazni, the capital city of Ghazni Province, were investigated in this study during the period November 2013–April 2014. The examined students were representative of children inhabiting eastern Afghanistan. Stool samples collected from the children were placed in sterile plastic vessels, fixed in 10% formalin and transported to the Military Institute of Medicine in Poland, where they were investigated microscopically for the presence of intestinal parasites (Korzeniewski et al. 2016). Simultaneously, a part of each sample was placed in a 2-mL sterile tube and analysed at the Department of Tropical Parasitology, Medical University of Gdańsk, Poland, using molecular detection methods as described below. Each faecal sample was kept in formalin up to 4 weeks before being subjected to cleaning and molecular investigations.

### Protocol used for the detection of *G. intestinalis* in formalin-fixed stool samples

#### Washing of stool samples

First, to remove the formalin solution, each stool sample was centrifuged for 10 min at 2500×*g* and the obtained supernatant carefully removed. Next, the sample was washed five times with sterile water as follows: 0.6 g of faeces was placed in a new sterile 2-mL tube, which was then filled with sterile water; the faeces were mixed in an automatic vortex for 20 s and centrifuged for 3 min at 2500×*g*; the obtained supernatant was removed with a pipette. The resulting pellet was stored at −20 °C for further analysis.

#### DNA extraction

Prior to the DNA extraction, the final pellet obtained after washing was frozen three times at −70 °C and thawed at 30 °C in a water bath to disrupt the cyst walls and to improve the efficiency of DNA extraction. Isolation of DNA was performed with the use of a Genomic Mini AX Stool kit (A&A Biotechnology, Gdynia, Poland). For the series of 0.1-g faecal samples, extraction was performed according to the original protocol. However, for the series of 0.6-g faecal samples, we introduced minor modifications to the manufacturer’s instructions. Specifically, each faecal sample was mixed with LS buffer and 40 μL of proteinase K and incubated for 45 min at 50 °C. The subsequent steps were consistent with the original protocol. All of the PCR templates were treated with an Anty-Inhibitor Kit (A&A Biotechnology, Gdynia, Poland), which removed polyphenolic PCR inhibitors using specific absorption particles, thereby removing factors that could interfere with PCR. The PCR templates were stored at −20 °C.

#### Detection of *G. intestinalis* DNA

For specific detection of *G. intestinalis* DNA in two series of *Giardia* cysts contained in formalin-fixed stool samples, we used two molecular methods involving different lengths of PCR products: nested PCR and real-time PCR, developed by Hopkins et al. (1997) and Guy et al. (2003), respectively.

All faecal samples collected from Afghan children were investigated with real-time PCR only.

#### Detection with real-time PCR

Real-time PCR was performed with the use of the β-giardin P241 primer TaqMan probe set specific to a 74-bp fragment of the *G. intestinalis* β-giardin gene (Guy et al. 2003). The amplification reaction mixture consisted of 12.5 μL of real-time 2× HS-PCR Master Mix Probe (A&A Biotechnology, Gdynia, Poland), 300 nM of each primer (Metabion, Germany), 200 nM of the hydrolysis probe (Metabion, Germany) and 3 μL of template DNA in a 25 μL reaction volume. The amplification programme, consisting of initial denaturation (10 min at 95 °C), 45 cycles of denaturation (15 s at 95 °C) and annealing and elongation (1 min at 60 °C), was performed in an Mx3005P thermocycler (Stratagene, USA). PCR products were analysed using MxPro QPCR software. The cycle threshold (CT) value, determining the cycle number at which the reporter’s fluorescence exceeded the threshold value, was recorded.

#### Detection with nested PCR

Nested PCR was performed with the use of a set of the primers GIAF, GIAR (outer primers enclosing 497-bp product) and RH4 and RH11 (inner primers enclosing 292-bp product) specific to the small subunit (SSU) ribosomal RNA (rRNA) gene of *G. intestinalis* developed by Hopkins et al. (1997). The amplification reaction mixture consisted of 12.5 μL of the standard and ready-to-use PCR mixture 2× PCR Mix Plus High GC (A&A Biotechnology, Poland) containing recombinant Taq polymerase, PCR buffer, magnesium chloride, nucleotides, stabilisers, gel loading buffer, 0.25 μM of each primer (Metabion, Germany) and 3 and 1 μL of template DNA for the first and second PCR rounds, respectively, in a 25 μL reaction volume. Amplifications were performed with an initial polymerase activation step (10 min at 95 °C), followed by 35 cycles of denaturation (45 s at 96 °C), annealing of primers (30 s at 55 °C for the outer and 5 s at 9 °C for the inner PCR), strand extension (30 s at 72 °C) and final extension (4 min at 72 °C). Nested PCR reactions were performed using a GeneAmp PCR System 9700 Thermal Cycler (Applied Biosystems, USA). The PCR products (292 bp) were analysed using a GelDoc-It Imaging System (UVP, USA) following electrophoresis on a 2% gel agarose, which was stained with Midori Green DNA Stain (Nippon Genetics Europe GmbH, Germany).

All experiments were performed including *Giardia*-positive controls (genomic DNA extracted from trophozoites of a *Giardia* strain cultured axenically in the Department of Tropical Parasitology, Medical University of Gdańsk, Poland) to ensure the correct functioning of the reaction and negative controls (water template) to control contamination of the PCR components.

#### Identification of Giardia assemblages A and B using real-time PCR

All positive *Giardia* faecal samples collected from Afghan children were genotyped. Real-time PCR was carried out on an Mx3005P thermocycler (Stratagene, USA) with the *Giardia*-specific β-giardin primer probe sets P434 (P-1), designed based on the Portland 1 sequence of the *Giardia lamblia* β-giardin gene (assemblage A), and P434 (H3), designed based on the H3 sequence of the *G. lamblia* β-giardin gene (assemblage B), according to Guy et al. (2003, 2004). The amplification reaction mixture and programme were identical with those used for the β-giardin P241 primer TaqMan probe set described above.

## Results

### Detection of *G. intestinalis* using molecular methods depending on duration of fixation in formalin

**Fig. 1:**
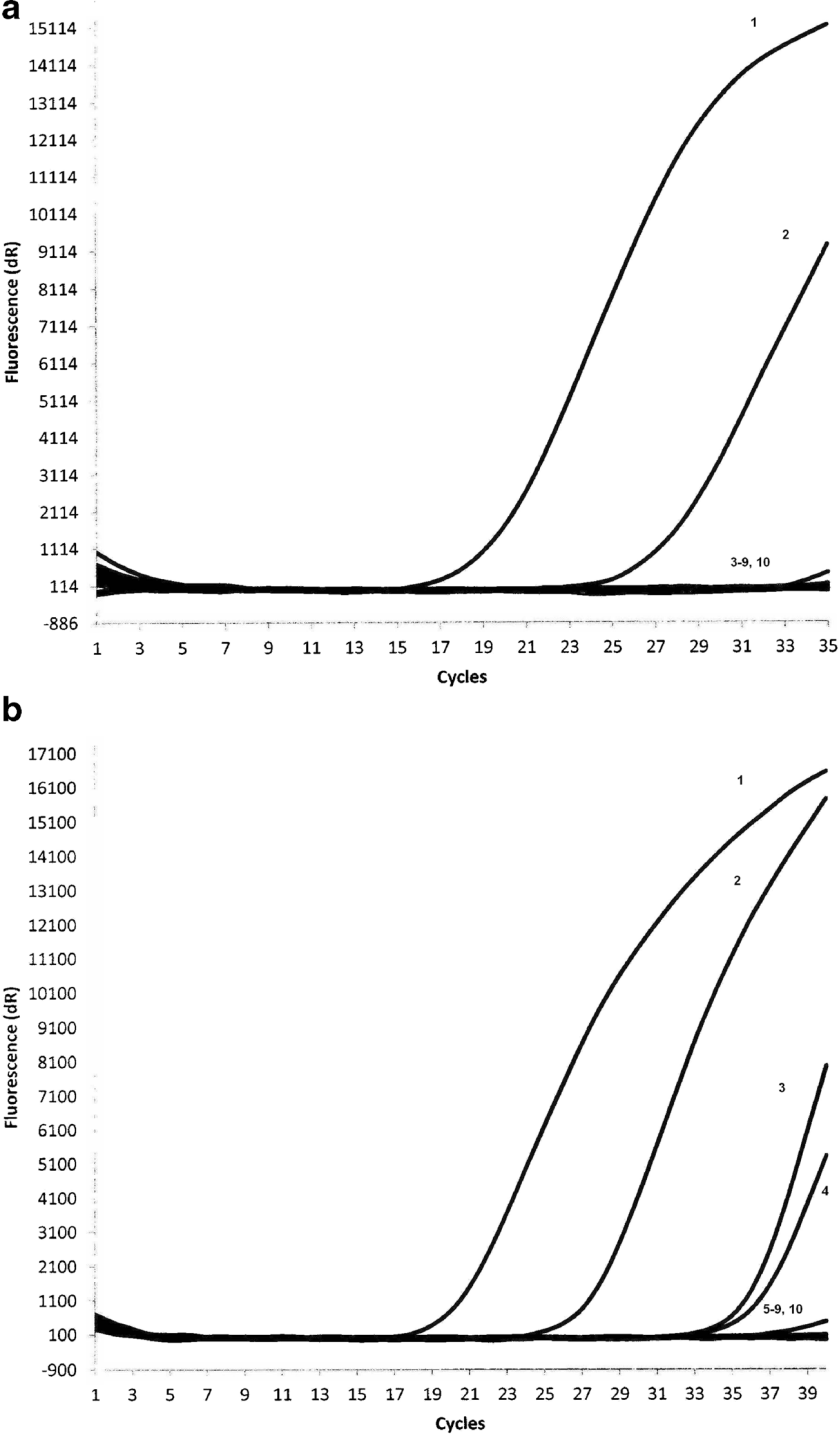
Detection of DNA of *G. intestinalis* from faeces containing *Giardia* cysts fixed in formalin using real-time PCR. **a** 0.1 g samples: *Giardia-*positive control (*line 1*), positive sample kept in formalin for 2 weeks (*line 2*), negative samples kept in formalin over 4 weeks (*lines 3–9*) and negative control (*line 10*). **b** 0.6 g samples: *Giardia-*positive control (*line 1*), positive sample kept in formalin for 2 weeks (*line 2*), positive sample kept in formalin for 4 weeks (*line 3*), positive sample kept in formalin for 6 weeks (*line 4*), negative samples kept in formalin over 6 weeks (*lines 5–9*) and negative control (*line 10*)

**Fig. 2:**
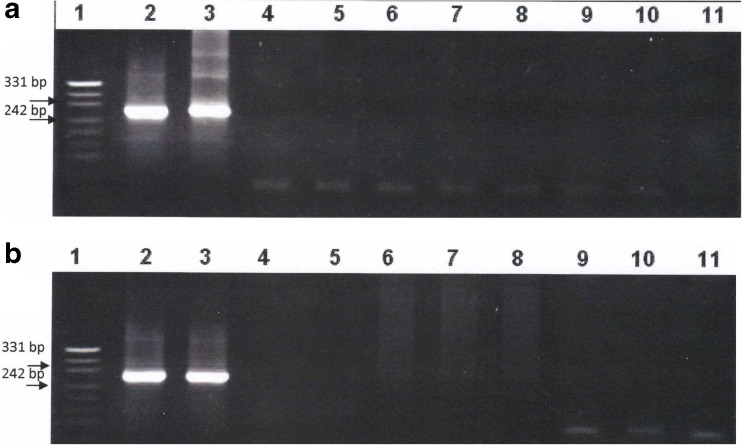
Detection of *G. intestinalis* from faeces containing *Giardia* cysts fixed in formalin using nested PCR. **a** 0.1 g samples. **b** 0.6 g samples: molecular weight marker (pUC19 DNA/MspI (HpaII) Marker, Thermo Scientific) (*line 1*), *Giardia-*positive control (*line 2*), positive sample kept in formalin for 2 weeks (*line 3*), negative samples kept in formalin over 4 weeks (*lines 4–10*) and negative control (*line 11*)

Two series of eight formalin-fixed stool samples (0.1 and 0.6 g) were investigated with the use of nested PCR and real-time PCR assays. In the case of the series with the smaller volume of samples, we obtained positive results from real-time PCR and nested PCR only for samples kept in formalin for 2 weeks (Table 1, Figs. 1a and 2a). In the case of the other series of samples with greater volumes, DNA of *G. intestinalis* was detectable up to 6 weeks after fixation in formalin using real-time PCR and 2 weeks using nested PCR (Table 1, Figs. 1b and 2b). When a *G. intestinalis* reference DNA was added to the negative samples in both series, amplification was observed in all samples tested.

**Table 1:** Results of detection of DNA of *G. intestinalis* using real-time and nested PCR in formalin-fixed stool samples containing *Giardia* cysts depending on the time of fixation in formalin and sample size

Detection method	Sample size	Detection of *G. intestinalis* DNA in correlation to the fixation time (weeks) in formalin							
		1	2	3	4	5	6	7	8
Real-time PCR	0.1 g	+	−	−	−	−	−	−	−
	0.6 g	+	+	+	−	−	−	−	−
Nested PCR	0.1 g	+	−	−	−	−	−	−	−
	0.6 g	+	−	−	−	−	−	−	−

Two weeks in formalin (*1*), 4 weeks in formalin (*2*), 6 weeks in formalin (*3*), 8 weeks in formalin (*4*), 10 weeks in formalin (*5*), 12 weeks in formalin (*6*), 14 weeks in formalin (*7*), 16 weeks in formalin (*8*)

*+* positive result of real-time PCR, − negative result of real-time PCR

### Results of the investigation of *G. intestinalis* detection in the Afghan children

**Fig. 3:**
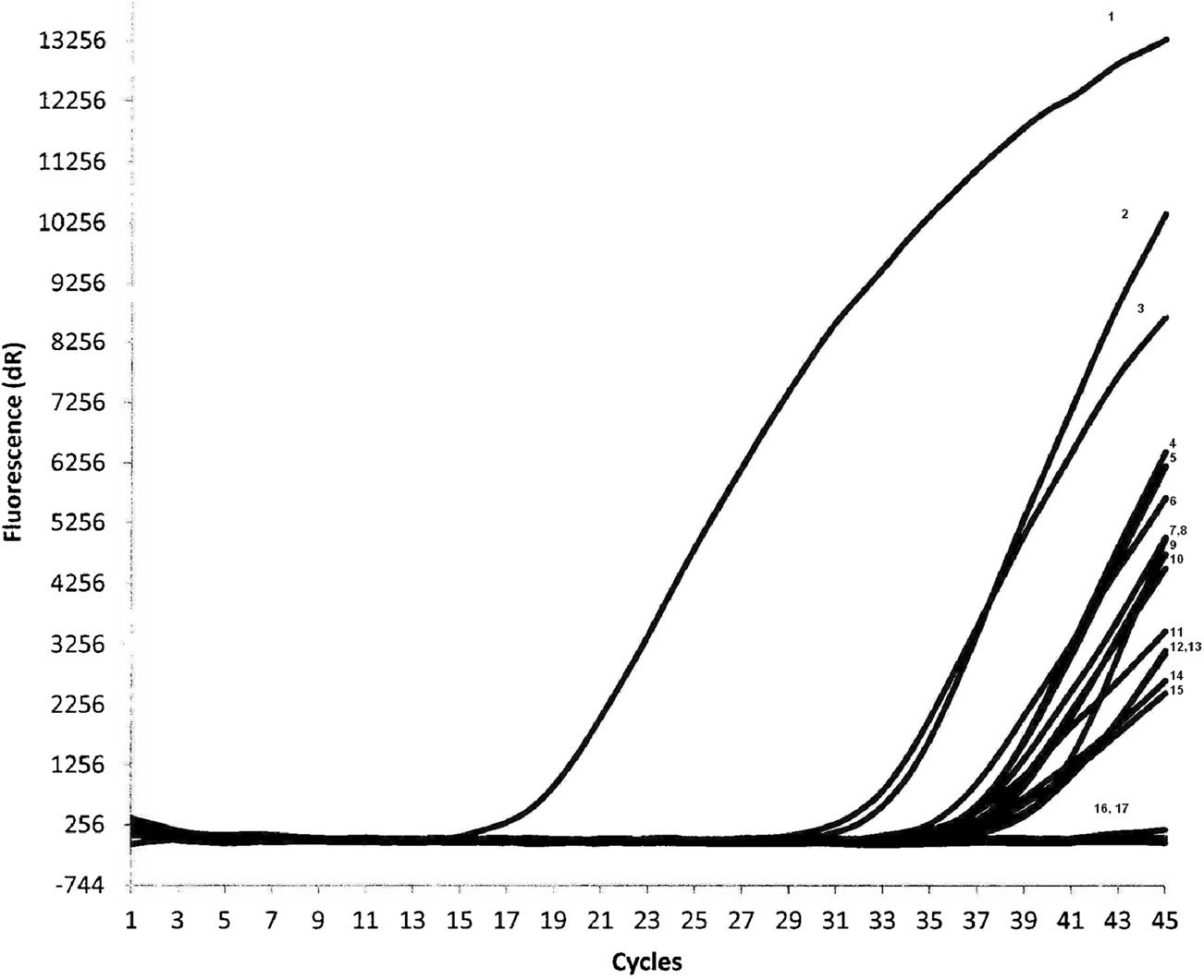
Results of detection of *G. intestinalis* DNA in faecal samples collected from Afghan children using real-time PCR; real-time PCR performed for first 94 templates investigated in this study. *Giardia*-positive control (*line 1*), positive samples (template nos. 1–3, 5–15; Table 2) (*lines 2–15*), negative samples (*line 16*) and negative control (*line 17*)

Among 245 faecal samples collected from Afghan children, DNA of *G. intestinalis* was detected in 52 (21.2%) samples using real-time PCR (Table 2, Fig. 3). The majority of infected children (of individuals with the age estimated) were 5–12 years old (Table 2).

**Table 2:** Detection of *G. intestinalis* DNA using real-time PCR in faecal samples collected from Afghan children in comparison to microscopic investigation (Korzeniewski et al. 2017)

Template no.	Age of the children	Microscopy (Korzeniewski et al. 2017)	Real-time PCR		
			*Giardia*	A	B
1	nd	+	+	−	−
2	nd	+	+	+	−
3	nd	+	+	+	−
4	nd	+	−	−	−
5	nd	+	+	−	+
6	9	+	+	−	+
7	nd	+	+	−	+
8	12	−	+	−	−
9	10	+	+	+	−
10	10	−	+	−	−
11	12	+	+	+	−
12	10	+	+	−	−
13	nd	+	+	+	−
14	nd	−	+	+	−
15	nd	+	+	−	+
16	nd	+	+	−	+
17	nd	+	+	+	+
18	nd	+	+	+	−
19	nd	+	+	+	−
20	nd	+	+	+	+
21	nd	+	+	−	+
22	9	+	+	−	+
23	nd	+	+	−	+
24	nd	+	+	+	+
25	nd	−	+	−	+
26	10	−	+	−	−
27	10	+	+	−	−
28	9	+	+	+	−
29	8	+	−	−	−
30	10	−	+	−	−
31	8	+	+	−	+
32	10	+	+	+	+
33	8	+	+	−	−
34	8	−	+	−	−
35	10	+	−	−	−
36	10	+	+	−	+
37	11	+	+	−	−
38	10	+	−	−	−
39	11	+	+	−	−
40	5	+	+	−	−
41	18	+	+	−	+
42	12	+	+	−	+
43	10	+	−	−	−
44	12	−	−	−	−
45	11	−	+	−	−
46	10	+	+	−	−
47	10	+	+	−	+
48	8	−	+	−	−
49	10	+	−	−	−
50	12	+	+	−	+
51	10	−	+	−	+
52	9	+	+	−	+
53	18	−	+	−	−
54	11	+	+	−	+
55	nd	−	+	−	+
56	13	−	+	+	−
57	15	−	+	−	+
58	16	+	+	−	−
59	19	+	+	+	−

*nd* not determined, *+* positive results, *−* negative result, *A* assemblage A, *B* assemblage B

Genotyping was successful in 39 positive faecal samples, revealing the presence of *G. intestinalis* assemblage A in 15 and assemblage B in 24. In four samples, both assemblages were detected. A comparison of this data with the results of a microscopic investigation performed earlier (Korzeniewski et al. 2017) is shown in Table 2.

## Discussion

Afghanistan is one of the poorest countries in the world, according to UNDP classification (rank 169/188 countries), based on wealth and economic development level (United Nations Development Programme 2016). The country is third in terms of the death rate of the whole population (17.65/1000 inhabitants die every year); one in five Afghan children dies before their fifth birthday (Central Intelligence Agency, World Factbook 2010). In terms of the occurrence of infective and invasive diseases, Afghanistan is classified as a high-risk country. This is due to contaminated water and soil, limited access to health services, a lack of basic drugs and medical equipment, a large number of asymptomatic carriers of infective and parasitic diseases among local populations and mass migration (Korzeniewski 2009; Wallace et al. 2002). Contamination of water with pathogenic agents is common; only 31% of Afghan households have access to clean drinking water. Moreover, only 5–7% of the Afghan population has access to basic standard toilets (United Nations Environment Programme 2009). The low awareness of hygiene and disease prevention in Afghan society exacerbates the situation. Afghanistan is a country associated with the Organisation of Islamic Cooperation (consisting of 57 countries), for which there is no data about the occurrence of intestinal parasitic infestations in the community (Hotez 2009). Data on the prevalence of intestinal parasitic infections among people living in Afghanistan is very limited. Studies performed by the scientists from different international military medical services in 2002 and researchers from WHO in 2003 showed high rates of parasitic infections among inhabitants of Afghanistan (Scheid and Thoma 2004; Gabrielli et al. 2005). More recently, our team performed microscopic examinations for intestinal parasitic infections on a large number of inhabitants, especially schoolchildren, of Ghazni Province (Korzeniewski et al. 2014, 2015a,b, 2016, 2017). Faecal material was collected by Polish medical staff and diagnosticians working at the Forward Operating Base in Ghazni, with the aim of detecting and controlling intestinal parasitic infections in the local population.

In this paper, we present the results of molecular investigations of formalin-fixed stool samples collected from 245 students of the Share Kona and Khuija Ali high schools in the city of Ghazni, Ghazni Province, Afghanistan. Using real-time PCR, we detected DNA of *G. intestinalis* in 21.2% of samples. Results of microscopic investigations performed earlier (Korzeniewski et al. 2017) on this group of children showed a prevalence of this parasite of 17.9%. Of the investigated samples, 38 positive samples were confirmed by both methods applied. DNA amplification was additionally successful in 14 samples that tested negative in microscopic investigations. In five samples, *Giardia* cysts were identified by microscopy, but were not confirmed by real-time PCR (Table 2). Our results confirmed a high rate of *Giardia* infections in schoolchildren in Afghanistan. Genotyping of positive samples showed the predominance of assemblage B of *G. intestinalis* in this population.

Genotyping of *Giardia* isolates from humans performed in various countries and on various continents showed the occurrence of a higher percentage of either genotype A or B. The predominance of genotype B has been demonstrated in several studies. For example, Molina et al. (2007) found genotype B in all human faecal samples (*n* = 34) collected from residents of the rural community of General Mansilla in Buenos Aires Province, Argentina, which tested positive for *G. lamblia*, using microscopy. Another study, performed by de Lucio et al. (2015), demonstrated that assemblage B was the most prevalent in patients with clinical giardiasis in central Spain. In north-western England, the majority of infections (64%) were caused by assemblage B in *Giardia* symptomatic patients, as reported by Minetti et al. (2015). The predominance of assemblage B was also recorded in human faecal samples in Egypt (Foronda et al. 2008); however, a predominance of genotype A was recorded, for example, in Turkey (Tamer et al. 2015) and in south-eastern Mexico (Torres-Romero et al. 2014). In our study, amplification of DNA fragments referring to genotypes (A and B) typically found in humans was successful in 39 of 52 positive samples. The negative results in 13 samples (25%) may have been due to the presence of local genotypic variants of the β-giardin gene of *G. intestinalis*, which could not be detected by the primers used. Another explanation may be that other genotypes, for example, assemblage E, may be present in this population. However, these aspects require further clarification.

We demonstrated that real-time PCR was able to detect DNA of the parasite, even though sample faeces had been conserved in 10% formalin for about 1 month. Fixatives are essential for the transportation and preservation of stool specimens. However, the preservation of stool samples in 10% buffered formalin (a traditional, commonly used stool fixative) is reported to hamper product amplification via PCR. Faecal samples for molecular method diagnosis are generally collected without preservatives because the use of fresh faeces prevents false negative results (Troll et al. 1997; Dowd et al. 1998). Inhibition of PCR and reduction of its sensitivity are probably the consequences of DNA fragmentation caused by formalin treatment (Ohara et al*.* 1992; Honma et al*.* 1993). The negative influence of formalin on various stages of intestinal parasites was reported by, e.g. Ramos et al. (1999), who showed that preservation of stool samples in 10% buffered formalin for more than 7 days hampered successful PCR amplification of DNA of the trophozoite *Entamoeba histolytica*. Contrastingly, Paglia and Visca (2004) demonstrated results of a study in which nested PCR, with initial amplification of the 1076-bp fragment of the SSU rRNA gene, had been applied for the specific detection of *E. histolytica/Entamoeba dispar* in faeces fixed in 10% formalin for 90 days. According to the authors, the extended time of contact of the specimens with formalin fixative had no apparent influence on PCR results. In our studies, the samples intended for copro-parasitic examination could not be investigated immediately following collection; thus, the use of formalin as a preservative was necessary in order to transport the material from Afghanistan to Poland. Examination of these samples in the laboratory was possible no earlier than 3–4 weeks after their collection. Several authors have suggested ensuing purification treatments to remove inhibitors from faecal samples (Amar et al. 2003; Guy et al. 2003; Homan and Mank 2001; Molina et al. 2007). In this study, the inhibiting substances present in faeces were removed by preisolation washing steps, as well as after extraction of DNA with the use of a commercial Anty-Inhibitor Kit. Nevertheless, the detection test showed that amplification was not possible in samples preserved in formalin for more than 6 weeks by real-time PCR and 2 weeks by nested PCR. The failures of amplification most probably derived from the low quality of the sample DNA, caused by its degradation over time and/or possible modifications caused by several substances, including formalin (Dowd et al. 1998; Troll et al. 1997; Wilson 1997). In our experiments, when reference DNA was added to the PCR negative templates (inhibition control), amplification was obtained in all of the samples. This suggests that the added DNA was of better quality than the DNA obtained from samples preserved in formalin for a long time and confirms the negative influence of this fixative on DNA. Al-Soud and Rådström (2000) suggested that adding DNA of better quality or in higher quantities may help to reduce the effect of the inhibitors present in the sample. Another possible explanation for problematic amplification is the presence in the samples of an amount of DNA insufficient to counteract the effect of the inhibitors, as suggested by Ghosh et al. (2000). Our tests showed that when DNA was isolated from 100 mg of the stool (a standard amount for a commercial DNA extraction kit) using the standard procedure, we were able to detect the DNA of the parasite after preservation in formalin no longer than 2 weeks. However, a greater volume of faeces and appropriate adjustment of the DNA extraction protocol resulted in positive real-time PCR results obtained for samples kept in formalin up to 6 weeks and in nested PCR for 2 weeks. The visible advantage of real-time PCR was most probably due to amplification of a much shorter product (74 bp) than in the case of nested PCR (497 bp in first-round PCR), which may be significant when degradation of DNA occurs in the sample.

This study, which constitutes the first description and genotyping of *G. intestinalis* in formalin-fixed stool samples collected from children in eastern Afghanistan, confirms the presence of assemblages A and B of pathogenic types of *Giardia*. The study provides useful information on the state of preservation of faecal material for delayed examination, detection and characterisation of genetic strains of pathogenic intestinal parasites like *Giardia*. It brings into focus certain questions about the exposure of schoolchildren to gastrointestinal parasites, which should be considered in the appropriate targeting of health education control programmes to prevent giardiasis and other intestinal infections in schoolchildren in Afghanistan.
